# SEX DIFFERENCES IN THE ASSOCIATION BETWEEN COMORBIDITIES AND COGNITIVE STATUS IN OLDER BLACK AMERICANS

**DOI:** 10.1093/geroni/igae098.4074

**Published:** 2024-12-31

**Authors:** Albert Junior Nyarko, Darlingtina Esiaka

**Affiliations:** University of Kentucky, Lexington, Kentucky, United States; University of Kentucky College of Medicine, University of Kentucky, Kentucky, United States

## Abstract

The decline in cognitive status among aging populations is a growing concern, particularly for Black Americans who experience a higher burden of chronic conditions that are known precursors of cognitive impairment. Despite the known disparities in brain health outcomes among aging Black Americans, limited research has explored how sex interacts with comorbid conditions to impact cognitive status in this population. We investigated sex differences in the association between comorbidities (stroke, diabetes, hypertension, sleep apnea, PTSD, active depression, cancer, asthma, and brain tumors) and cognitive status (presence of abnormal cognitive status) among older Black Americans. We analyzed data from participants (N= 448, 330 females; Mage = 75.79, SDage = 7.40; mean years of education = 15.89) who are a part of the University of Kentucky ADRC cohort. Our analysis showed significant sex differences in the association between comorbidities and cognitive status. Having a higher number of comorbidities was associated with greater presence of abnormal cognitive status in Black American males (b =.016, p =.131) than females (b =.002, p =.580). Further analysis showed that hypertension was significantly associated with a greater presence of abnormal cognitive status in Black American males (b =.295, p =.007). The presence of comorbidities, especially hypertension, may impact cognition more in older Black American males than females. Our findings highlight the complexity of comorbidities’ impact on cognition and underscore the importance of considering sex differences in research on aging and brain health in Black Americans.

